# Data Storage Mechanism Based on Blockchain with Privacy Protection in Wireless Body Area Network

**DOI:** 10.3390/s19102395

**Published:** 2019-05-25

**Authors:** Yongjun Ren, Yan Leng, Fujian Zhu, Jin Wang, Hye-Jin Kim

**Affiliations:** 1 School of Computer and Software, Nanjing University of Information Science & Technology, Nanjing 210044, China; renyj100@126.com (Y.R.); 18068857497@163.com (Y.L.); zhufujian1995@gmail.com (F.Z.); 2 Jiangsu Collaborative Innovation Center of Atmospheric Environment and Equipment Technology (CICAEET), Nanjing University of Information Science & Technology, Nanjing 210044, China; 3 School of Computer & Communication Engineering, Changsha University of Science & Technology, Changsha 410004, China; jinwang@csust.edu.cn; 4 School of Information Science and Engineering, Fujian University of Technology, Fujian 350118, China; 5 Business Administration Research Institute, Sungshin Women’s University, Seoul 02844, Korea

**Keywords:** wireless body area network, data storage, blockchain, digital signature

## Abstract

Wireless body area networks (WBANs) are expected to play a vital role in the field of patient-health monitoring shortly. They provide a convenient way to collect patient data, but they also bring serious problems which are mainly reflected in the safe storage of the collected data. The privacy and security of data storage in WBAN devices cannot meet the needs of WBAN users. Therefore, this paper adopts blockchain technology to store data, which improves the security of the collected data. Moreover, a storage model based on blockchain in WBAN is proposed in our solution. However, blockchain storage brings new problems, for example, that the storage space of blockchain is small, and the stored content is open to unauthorized attackers. To solve the problems above, this paper proposed a sequential aggregate signature scheme with a designated verifier (DVSSA) to ensure that the user’s data can only be viewed by the designated person and to protect the privacy of the users of WBAN. In addition, the new signature scheme can also compress the size of the blockchain storage space.

## 1. Introduction

Wireless body area network (WBAN) is an underlying technology that can monitor and record human health signals for a long time. Its early application is mainly used to continuously monitor and record health parameters of patients with chronic diseases (such as diabetes, asthma, and heart disease) and provide some form of automatic therapy control. For example, once a diabetic’s insulin level drops, the WBAN in their body will immediately activate a pump that automatically injects insulin into the patient, which allows the patient to keep insulin at a normal level without a doctor [1,2,3]. In the future, it can be widely used in consumer electronics, entertainment, sports, environmental intelligence, ubiquitous computing, military, or security fields. Not only these applications but also the so-called “smart dust” (microscopic devices with processing power and wireless communications), which is currently stuck in the realm of science fiction, is entirely possible in the future [4,5,6].

Wireless body area network in the world has been widely studied, including medical technology providers, hospitals, and insurance companies, as well as the industry parties that are carrying out strategic cooperation. WBANs have become a very popular research topic and are applied to many applications. They provide pervasive computing services and techniques in various potential applications for the internet of things (IoT) [7,8]. However, WBAN is still in its early stage, which faces challenges in the milliwatts level network energy consumption, interoperability, system equipment, security, sensor validation, data consistency, and so forth. The IEEE802.15 task group completed the world’s first WBAN standard, IEEE 802.15.6, in 2012. In 1998, the IEEE 802.15 working group was established to specialize in wireless personal area network (WPAN) standardization. Its mission was to develop a standard for short-range wireless communications, a wireless personal area network (WPAN), as it is commonly called. The technology is a major health care breakthrough when adopted [9,10].

Although the wireless body area network brings great conveniences, it also brings some hidden dangers. As WBAN stores and processes personal health information (e.g., health, history, vital signs, etc.), it raises several privacy and safety concerns [11,12,13,14,15]. In general, two types of threats exist [16]:

(1) Unauthorized access: The unauthorized attackers hack into the WBAN and steal user data. Such attacks will violate users’ privacy, for example, if the attacker sells users’ information to an insurance company.
(2) Tampering with the messages: The attacker modifies signals in the WBAN so that the data collector receives fake users’ data. This will affect the safety of users, for example, if the user is a patient, and the patient data received by the doctor is false data, which would lead to the wrong treatment by the doctor.

For the above two threats, this paper adopts blockchain technology and a particular digital signature to solve these problems. Our contribution is the following two points:

- Blockchain: We use blockchain to store the WBAN user’s data which can prevent the data from being tampered with.
- The DVSSA scheme: We propose a sequential aggregate signature scheme with a designated verifier. It ensures that the user’s data can only be viewed by the administrator, and in other hands it can be compressed to the size of the blockchain storage space, which solves the illegal access problem.

We organized the rest of our paper as follows. Section 2 first introduces the basic knowledge of WBAN and then introduces the blockchain structure and characteristics. Section 3 presents the security requirements of WBAN. In Section 4, our system model is shown. Section 5 presents our DVSSA scheme. In Section 6, the DVSSA scheme in blockchain and the energy consumption for message computation and transmission are evaluated. Finally, our paper is concluded in Section 7.

## 2. Related Work

### 2.1. Wireless Body Area Network

#### 2.1.1. Network Architecture

The WBAN network architecture is an important part of the system architecture. It is the logical organization of communication devices (such as sensor nodes) in the system. Common network architectures include star topology, mesh topology, ring topology, and bus topology. The choice of network architecture is affected by the characteristics of the system and can affect many aspects of the system’s performance, such as energy consumption, traffic load handling capacity, node failure robustness, and MAC (Media Access Control Address) protocol selection. The purpose of choosing the WBAN network architecture is to better ensure low energy consumption and reliable data transmission of wireless communication. The selection of the architecture needs to consider the following factors: energy consumption, transmission delay, inter-user interference, node failure, and mobility [17,18,19].

In general, the star topology network structure corresponds to a one-hop wireless communication mode, while a mesh topology structure corresponds to a multiple-hop wireless communication mode. Traditional WBAN network topologies generally use simple star topologies, but there may be network or mixed topologies. For example, when the nodes are far away from the body or blocked by the body, the multi-hop communication mode is required. The choice of network architecture is not a single-hop communication mode. From the perspective of the practical application, in general, the size and complexity of the WBAN network are the main basis for architecture selection, for example, the WBAN physiological data acquisition system for monitoring patient health, the WBAN application architecture for a healthcare system based on ultra-bandwidth communication, or the WBAN system for realizing low energy consumption or a lightweight wireless communication protocol. In contrast, for a WBAN network with many nodes or of a large scale, the mesh topology or mixed topology should be selected.

WBAN networks with a network or mixed topology structure have more research value, and there is a lot of work to be done on such networks. On the one hand, the probabilistic connection model is proposed in multi-hop WBAN networks instead of the circular coverage model to solve the wireless communication connection problem. Furthermore, in a multiple-hop communication architecture, there may not be various communication links between the two entities. Thus, the corresponding mesh topology, which requires more complex multi-hop communication, will also facilitate the wearable sensors and sensors around them and recycle distributed reasoning methods or strategies to implement intelligent identification and monitoring. Larger multi-hop wireless communication is another possible role for the WBAN network control system [20,21,22,23].

#### 2.1.2. Wireless Communication Technique in WBAN

According to the position of the radio signal, the communication in the WBAN includes two kinds: in vivo communication and in vitro communication. Body-coupled communication is a new method for in vivo communication, using the human body as the transmission medium [24]. The literature [25] studied the relevant simulation method of in vivo communication. In the literature [26], this method is applied to identity recognition in the body domain network. In the WBAN, most devices are placed on the human body, so the external communication mainly refers to the short-distance and low-power communication around the human body. At present, the commonly used wireless communication technologies in WBAN include Bluetooth and IEEE802.15.4 (Zigbee) [27], ANT [28], Zarlink [29], and so forth. Zigbee technology is currently the most commonly used communication technology in the research of volume domain networks due to its characteristics of low speed, low power consumption, and low cost [30,31,32,33]. Furthermore, UWB (ultra-wide WBAN) communication technology, due to its characteristics of a high transmission rate, low cost, low power consumption, strong anti-interference ability, and strong multi-path resolution, has also been attracting more and more attention from scholars [34,35,36]. The IEEE launched 802.15.4 in 2007. The team [37] is responsible for the development of the body domain communication standard, the establishment of which will further promote the development of the WBAN.

#### 2.1.3. Management System and Database

As an integral part of the management of WBAN applications, the database is responsible for the sensory storage data collected by the WBAN network. A database management system can be used in a small choice of small-scale WBAN network DBMS (Database Management System, such as MySQL, Microsoft Access, and the Adaptive Server Anywhere) and medium-sized DBMS (e.g., Informix), as well as for mass WBAN networks of large-scale DBMS (such as DB2, Oracle, Sybase). Of course, text files can also be used to store data, such as the digital human pulse wave sample data stored in a local TXT (Plain text) file in the WBAN system of wireless traditional telemedicine. The database can adopt local centralized storage. In the WBAN system of wireless traditional telemedicine, the decoded and analyzed data can be connected to the specified database through the JDBC (Java DataBase Connectivity) interface, and a remotely distributed storage can also be adopted [37,38,39,40].

The management system can not only visualize the stored data but also manage the data, which includes the operations of adding, modifying, deleting, querying, and so forth. According to the requirements of the WBAN application, developers can choose the development tools and programming languages suitable for the management system, such as the Internet and local area network (LAN) ASP and JSP development environment or independent languages such as Delphi, Visual Basic. Development tools can include Python, C, C++ programming languages, etc. Developers can also choose monitoring functions and visual display interfaces in MATLAB to provide data analysis and intelligent processing. In the future, especially for the WBAN application of long-term continuous monitoring or the information-sharing platform built by multiple BSN (Body Sensors Network), considering the system performance and traffic demand, large amounts of data should be stored in a large distributed database for efficient management, and high-performance computing technology for these data should be developed in WBAN. Moreover, data can be further analyzed and processed (generating decisions, data mining, etc.).

### 2.2. Blockchain

#### 2.2.1. Structure of Blockchain

P2P (Peer-to-Peer) networks are responsible for ensuring the freedom of communication within blockchain nodes, which are geographically dispersed but have equally privileged participants in the application. There is no centralized server in the P2P network, and each node is an informed consumer and information provider. Each node participates in the routing process of the entire network, which is the discovery and maintenance of connections to neighboring nodes, the propagation and validation of transactions, and the synchronization of blocks of data (both transactions and blocks are data structures of the blockchain, as described below). This ‘flat’ topology of P2P networks is the key reflection of blockchain and the decentralized nature of the base. Blockchain applications provide APIs (application programming interfaces) for various scenarios. Users interact directly with them through these APIs without having to worry about the underlying technical details.

**Figure 1:**
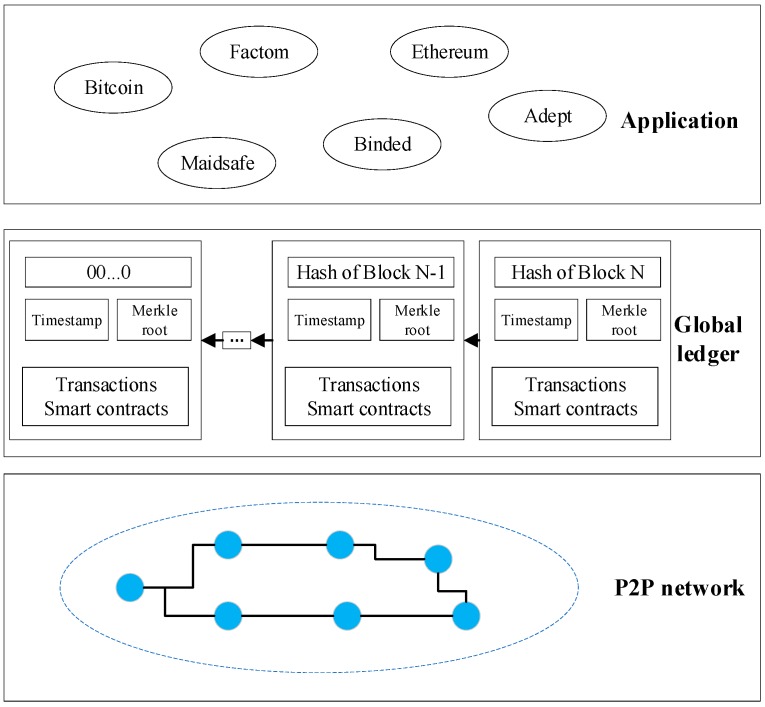
The basic structure of blockchain.

In general, a blockchain is an appended database, maintained by a peer-to-peer network node. As shown in Figure 1, the basic structure of blockchain can be divided into three levels, namely P2P network, database, and various applications.

#### 2.2.2. Key Characteristics of the Blockchain

From the research [41], we have summarized four attributes which describe a basic blockchain architecture as a general, decentralized ledger, offering data integrity and traceability. We describe these characteristics next.

(1) Autonomy: One important feature of blockchain is that there is no separate entity control or control network. In the public settings, any node can sign and publish transactions, and if they are accepted, the blockchain will check other nodes in their decentralized network at any time. In addition, everyone can join the consensus process to extend new blocks to the blockchain.
(2) Distributed: A blockchain system is built on a P2P network to which the source node broadcasts each signed single row of transactions. The adjacent peer then validates these incoming transactions: the valid transaction is forwarded further, and the invalid transaction is discarded. Eventually, these transactions can be extended to the entire P2P network. The system can process notifications and synchronizes networks for newly generated blocks.
(3) Non-tampering: All valid blocks and transactions recorded in the global ledger are virtually immutable due to the need for validation by other nodes and traceability of changes. Furthermore, the entire global ledger is synchronized between blockchain nodes according to a consensus mechanism, giving users greater confidence in the authenticity and accuracy of the data in the blockchain.
(4) Contractual: The process of consensus (for example, mining or voting) depends on the state of the data in question. The consensus is reached through the implementation of rules, i.e., the blockchain of the smart contract, for example, does not have any central authorization. The rules defined by these codes ensure that actions in any currency are executed promptly and correctly without human intervention.

## 3. Problem Statement

### 3.1. Security Requirements of WBAN

The security elements of a WBAN consist of four main parts:

- **Data confidentiality:** In WBAN, data confidentiality is one of the most important problems; it can protect the user from data leaks. In medical applications, when the node collects and sends sensitive information to the coordinator, the enemy can eavesdrop on some key information in the communication, which will reveal the patient’s privacy. This kind of eavesdropping may bring serious damage to the patient. The traditional method is to encrypt the data and then retransmit to ensure the communication security of the external sensor node and the network coordinator, and only allow the receiver to be authorized to decrypt the WBAN node, but this is difficult to implement for the sensor node with poor computational performance.
- **Data integrity:** The confidentiality of the data does not guarantee that the data will not be tampered with. After the data is stolen by the opponent, it can be tampered or destroyed by adding or reducing data segments, and then the data will be sent to the network coordinator. Vital information can be compromised, which can be very dangerous to users. The data integrity mechanism ensures that the data transmitted between BSN and BSNC (Body sensors network coordinators) is not changed by the adversary. The sender uses a one-way algorithm to compute the MAC frame, generates the integrity code for the frame, and sends it attached to the packet. The receiver uses the same process to calculate the MAC frame and compares the calculated result with the one given by the sender, to judge whether the data was maliciously tampered with in the sending process.
- **Data authentication:** Data authentication is necessary for medical and non-medical applications. It enables the BSN and BSNC to verify that data is sent by trusted sensor nodes. This prevents hostile parties from sending false messages to trick BSN and BSNC data authentication.
- **Data freshness:** The freshness of the data can prevent the retransmission attack. The hostile party may capture the frame in the transmission process and resend the data after a period of delay to achieve the purpose of confusing the BSNC.

### 3.2. Possible Security Threats and Attacks on WBAN

WBAN is vulnerable to a significant number of attacks, which are carried out in different ways, such as denial-of-service attacks (DoS), privacy invasions, and physical attacks. Countering these attacks is challenging, as it is limited by the power consumption of sensor nodes. A robust sensor can easily block sensor nodes and prevent them from aggregating patient data.

The attacks on WBAN can be roughly divided into three types: (1) confidentiality and authentication attacks, in which the hostile party conducts eavesdropping and attempts reply attacks or electronic spoofing; (2) attack on service integrity; network forced to accept wrong information; (3) network availability attack and denial-of-service (DoS) attack affect network capacity and performance.

### 3.3. Security Solution for WBAN

For the security threats discussed in the previous section, Table 1 lists possible solutions: 

For patients in WBAN, the privacy of their own data is important. For example, patients do not want their data to be collected by insurance companies who can use the data for their purposes, such as selling patient information. In addition, the integrity and correctness of the patient data are also very important. If the data reviewed and analyzed by the manager (doctor or hospital) is incomplete or tampered, the medical judgment made by the doctor is likely to be wrong, which is extremely unsafe for patients. 

Thus, we propose the DVSSA signature scheme to solve the problem of unauthorized access in WBAN, so as to ensure that only the specified verifier can view and analyze the data of WBAN users. Moreover, we used blockchain to store WBAN user data to account for the problem of data tampering and guarantee the integrity of the data, based on the non-tamper property of blockchain. 

## 4. System Model

### 4.1. WBAN Model

**Figure 2:**
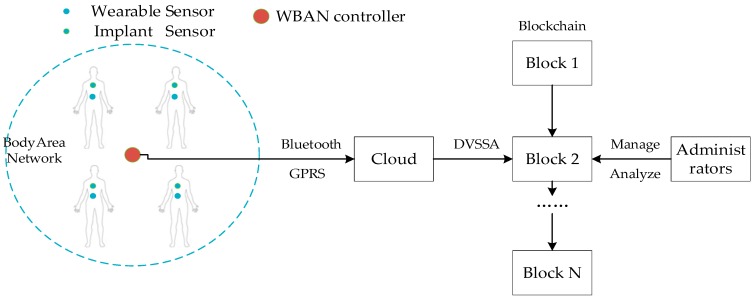
A wireless body area network (WBAN) architecture of a health care application.

We designed the WBAN system as shown in Figure 2. There are three main entities in this system:

(1) WBAN: the WBAN consists of a WBAN controller and several (implantable or wearable) devices. These devices are often sensors that monitor important body parameters or movements and control the body by providing life support, visual/auditory feedback, and so forth. The WBAN device communicates with the WBAN controller directly or through multi-hop communication. The WBAN controller communicates not only with the WBAN device but also with Cloud. Also, the close-range WBAN controller can form a self-organizing network using a wireless personal area network (WPAN) technology.
(2) External administrator: external entities are mainly doctors or hospitals that we called administrators. Administrators can view the data of WBAN users stored in the blockchain and manage and analyze the data.
(3) Cloud: cloud servers can provide the function of cloud storage service. Users can easily access data at anytime and anywhere through any internet-connected device connected to the cloud. More importantly, the cloud server cluster has a large number of storage resources, which can provide infinite storage space for the edge networks with limited resources.

The WBAN controller is used to send the collected user data to the cloud via Bluetooth or GPRS. After receiving the data, the cloud uses the DVSSA signature scheme to write the signed data into the blockchain. Administrators can view and analyze users’ data with their private keys.

### 4.2. Cloud-Blockchain Model

#### 4.2.1. The Advantage of Blockchain Storage

Traditional data storage solutions rely heavily on centralized databases to maintain security. For hackers, the targets are more specific. Once a hacker successfully executes a script attack on a centralized database, the hacker has access to a large amount of data. However, with blockchain and distributed ledger technology, cracking is much harder. Many blockchain projects aim to make data storage more secure. The potential benefit is ground-breaking for the end user. The blockchain project not only has the potential to create an architecture for inherently more secure data storage systems but also allows individual users to have full access to their data. In many cases, blockchain projects are using the original cryptocurrency as part of the markup model. This allow users to monetize any third-party data, while also preventing identity theft and other problems that have emerged in recent years due to large-scale data breaches. By using digital signatures, blockchain system transactions ensure the integrity and non-repudiation of messages.

#### 4.2.2. The Defect of Blockchain Storage

Blockchain is a data chain that is made up of multiple blocks, in which all transactions are stored. The blocks in the bitcoin blockchain were set initially to be 1M in size, but as the volume of transactions on the bitcoin blockchain has increased dramatically, 1M block has fallen far short of demand. The most direct way to solve this problem is block expansion, which is vulnerable to DDoS attacks (distributed denial of service) and thus has not been supported by the core development team of bitcoin. Furthermore, block expansion will significantly increase the cost of mining and cannot be supported by most mining pools. Hence, there are more and more digital signature schemes to compress the size of the blockchain.

#### 4.2.3. Our Solution

The DVSSA signature scheme proposed in this paper makes the size of the signature written into the blockchain equal to the size of a single person’s signature through the sequential aggregation of all people’s signatures, which greatly saves the storage space.

**Figure 3:**
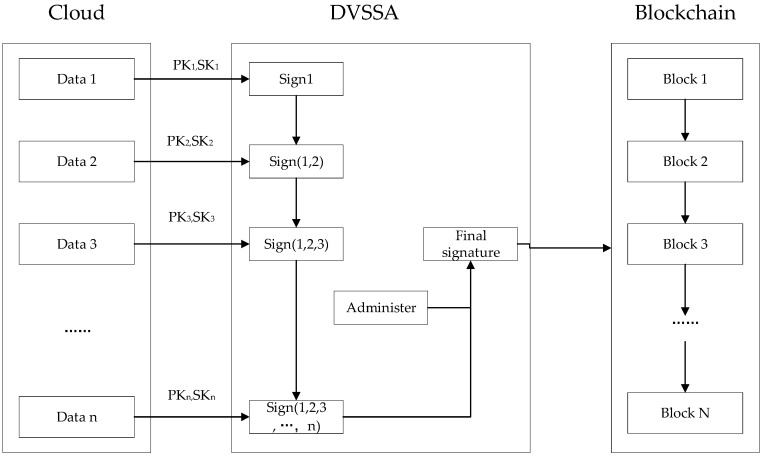
Data storage model.

The private data received by each user is stored in a different data block in the cloud, which is stored in the form of a linked list in the cloud. Then, the data of users in the cloud are signed through the DVSSA signature scheme and sent to the blockchain, as shown in Figure 3.

The WBAN controller sends the collected patient data to the cloud, which firstly divides the data of each patient into data_1_, data_2_…, data_n_, and each patient has its public key pair ($PK_{i}SK_{i}$) which is distributed by the administer. First, the first patient signs the corresponding data_1_ using his private key $SK_{1}$ and gets the signature sign_1_. Then, the second patient signs the data_2_ using his private key $SK_{2}$ and the first patient’s signature sign_1_ to get the signature sign_(1,2)_, and so forth to get the signature sign_(1,2..., n−1)_. Finally, the private key $SK_{n}$ of the n-th patient and the signature of the previous patient sign_(1,2..., n−1)_ sign the data_n_ to get the signature sign_(1,2..., n)_. We add the manager’s public key attribute to get the final signature and write it into the blockchain.

### 4.3. Data Validation Model

**Figure 4:**
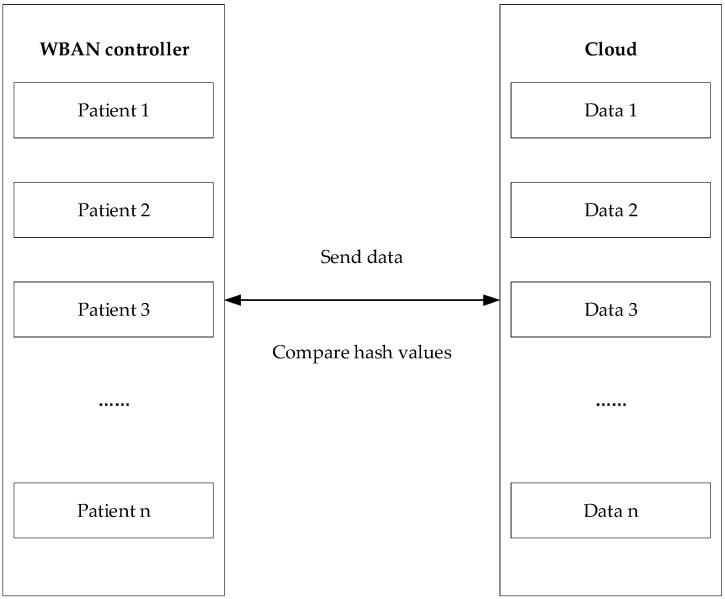
Data verification chart.

After the data are uploaded to the cloud server, we partition the data and write it into the blockchain using the DVSSA signature scheme. However, there are some security problems. How can we ensure that the data uploaded to the cloud is original data without any tampering? Therefore, we propose a data validation model as shown in Figure 4.

The WBAN controller hashes the blocks of data collected from each patient to get a hash value and stores it in the controller. Then each patient’s data is sent to the cloud server. The cloud server first blocks each patient’s data, then hashes each data block and returns the value compared with the hash value in the WBAN controller. If the value is the same, the next digital signature is performed. Otherwise the service is terminated.

## 5. Sequential Aggregate Signature with Designated Verifier

### 5.1. Preliminaries

#### 5.1.1. Bilinear Pairings

Let l be a security parameter, q is a prime order of l-bit, $G_{1}$ is a circulation additive group of the prime order q which is generated by P. $G_{2}$ is a circulation additive group of the prime order q, which is generated by Q. $G_{r}$ is a cyclic multiplicative group of prime order q. Our proposed DVSSAgg makes use of a bilinear map: $e:\;G_{1}$×$\;G_{2}$ → $G_{r}$, with the following properties:

- Bilinear: ∀$a,b\in Z_{q}^{*}$, there is $e\left(aP,bQ\right)=e{\left(P,Q\right)}^{ab}$.
- Non-degeneracy: e(P,Q)≠1.
- Computability: There is an efficient algorithm to compute e(P,Q).

In the above definition, $e:\;G_{1}$×$\;G_{2}$ → $G_{r}$ is an asymmetric bilinear pair if $G_{1}$ ≠ $G_{2}$; $\;e:\;G_{1\;}$×$\;G_{2}$ → $G_{r}$ is a symmetric bilinear pair if $\;G_{1}=G_{2}=G_{r}$, symmetric bilinear pairs can be regarded as special cases of asymmetric bilinear pairs. Bilinear maps e can be constructed by Weil pairs or Tate pairs on a hypersingular elliptic curve over a finite domain. 

#### 5.1.2. Bilinear Diffie-Hellman Problem (BDH)

Given two groups $G_{1}$ and $G_{2}$, with the same prime order q, let $e:\;G_{1\;}$×$\;G_{1}$ → $G_{2}$ be a bilinear map and g be a generator of $G_{1}$. The objective of BDH is to compute $e{\left(g,g\right)}^{abc}$ in $\left(G_{1},\;G_{2},e\right)$ from the given $\left(g,\;g^{a},g^{b},g^{c}\right)$, where $a,b,c\in Z_{q}$.

#### 5.1.3. Sequential Aggregate Signature Model

In the sequential aggregate signature, each signer must aggregate his signature into the current signature in a certain order. After each signer has signed, the aggregate signature is sent to the next signer, and the next signer can aggregate his signature only if they receive the aggregate signature. The specific steps are as follows:

**Step1 Setup (1^λ^):** input security parameter 1^λ^, output public parameter (Pa).

**Step2 KeyGen (*Pa*):** input public parameter Pa, output public key PK, and private key SK.

**Step3 AggSign(**$\sigma^{\prime }$**,**$\bar{M}$**,**$\bar{PK}$**,M,**SK**,**PK**,*Pa*):** input $\bar{M}=\left(M_{1},M_{2},\ldots \ldots M_{k}\right)$ and $\sigma^{\prime }$ and $\bar{PK}=\left(PK_{1},PK_{2},\ldots \ldots ,PK_{k}\right)$, the message $M_{k+1}$, private key SK, and public parameter Pa generate a new aggregate signature σ.

**Step4 AggVerify (**$\sigma ,\bar{M},\bar{\;PK},Pa$**):** input $\bar{M}=\left(M_{1},M_{2},\ldots \ldots M_{n}\right)$, aggregate signature σ, $\bar{PK}=\left(PK_{1},PK_{2},\ldots \ldots ,PK_{k}\right)$, and public parameter Pa, the output of a 1 or 0 indicates whether the signature is valid or invalid.

### 5.2. Our Scheme

**Step1 Setup(1^λ^):** First, generate a bilinear group G and $G_{r}$ of prime order p (length λ bit), $G=\langle g_{1}\rangle$, $G_{r}=\langle g_{2}\rangle$, randomly choose Y∈G, output public parameter $Pa=\left(P,G,G_{r},e,g,Y\right)$.

**Step2 SKeyGen (*Pa*):** Input public parameter *Pa*, randomly choose $x\in Z_{p}$, let $X=g_{1}{}^{x}$, output $SK_{A}=x$, $PK_{A}=X$.

**Step3 VKeyGen (*Pa*):** Input public parameter *Pa*, randomly choose $d\in Z_{p}$, let $D=g_{1}{}^{d}$, output $SK_{B}=d$,$\;PK_{B}=D$.

**Step4 DVSSAgg-Sign (**$\sigma^{\prime }\bar{M^{\prime }}$**,**$\bar{PK^{’}}$, M, $SK_{A}$**,**$PK_{B}$**, *Pa*):** Input message $\bar{M^{\prime }}=\left(M_{1},M_{2},\ldots \ldots M_{k}\right)$ and its aggregate signature $\sigma^{\prime }=\left(A^{\prime },B^{\prime },C^{\prime }\right)$, input $\;PK^{’}=\left(X_{1},X_{2},\ldots \ldots ,X_{k}\right)$ and public parameter is Pa. Input the Message $M_{k+1}$ (*M* is the last message) and its private key $SK_{A}$, and verifier’s public key $\;PK_{B}$ which generates a new sequential aggregation signature for the specified verifier $\sigma ={\left(A,B,C\right)}^{D}$. $A={\left(A^{\prime }\right)}^{’}$, $B={\left(B^{\prime }\right)}^{’}$, $C={\left(C^{\prime }{\left(A^{\prime }\right)}^{’}{\left(B^{\prime }\right)}^{xM}\right)}^{r}$.

**Step5 DVSSAgg-Verify (**$\sigma ,\bar{M},\bar{\;PK_{A}},SK_{B},Pa$**):** Input $\bar{M}=\left(M_{1},M_{2},\ldots \ldots M_{n}\right)$ and its sequential aggregation signature for the specified verifier $\;\sigma ={\left(A,B,C\right)}^{D}$, public chain is $\bar{\;Pthe\;K_{A}}=\left(X_{1},X_{2},\ldots \ldots ,X_{n}\right)$, check if the each public key $X_{i}$ appears only once in $\bar{\;PK_{A}}$. If it is, then verify whether the following formula is true:

$$e\left(A,Y\right)=e\left(B,g\right)\;e\left(C,g\right)=e\left(A,\prod_{i=1}^{n}X_{i}\right)\;\cdot \;e\left(B,\prod_{i=1}^{n}X_{i}{}^{M_{i}}\right)$$

If verified, the algorithm outputs 1, otherwise 0.

We can notice that the public key chain is $\bar{\;PK_{A}}=\left(X_{1},X_{2},\ldots \ldots ,X_{n}\right)$, in the sequential aggregation signature for the designated-verifier $\sigma ={\left(A,B,C\right)}^{D}$ about message chain $\bar{M}=\left(M_{1},M_{2},\ldots \ldots M_{n}\right)$, $A=g^{r}$, $B=Y^{r}$, $C={\left(g^{r}\right)}^{\sum_{i=1}^{n}X_{i}}\cdot {\left(Y^{r}\right)}^{\sum_{i=1}^{n}X_{i}M_{i}}$.

### 5.3. Security Proof

**Theorem** **1**. *The sequential aggregate signature scheme with a designated verifier generated by the signer with a valid signature algorithm must pass the validation algorithm*.

**Proof**. The correctness of the scheme is obvious, because:

$$e\left(A,Y\right)=e\left(g^{r},Y\right)=e\left(Y^{r},g\right)=e\left(B,g\right)$$

And:

$$e\left(C,g\right)=e\left({\left(g^{r}\right)}^{\sum_{i=1}^{n}X_{i}}\right){\cdot (Y^{r})}^{\sum_{i=1}^{n}X_{i}M_{i}},g)\;=e\left(g^{r},g^{\sum_{i=1}^{n}X_{i}}\right)\cdot e\left(Y^{r},g^{\sum_{i=1}^{n}X_{i}M_{i}}\right)\;=e\left(A,\prod_{i=1}^{n}X_{i}\right)\cdot \;e\left(B,\prod_{i=1}^{n}X_{i}{}^{M_{i}}\right)$$

 □

**Theorem** **2**. *If (*$t^{\prime },q^{\prime },\in^{\prime }$*) is unforgeable under G, we say DVSSAgg scheme*$\left(t,\;q_{C},\;q_{s},\;n,\;\in \right)$*is unforgeable, and *$t^{\prime }=t+O\left(q_{C}+nq_{s}+n\right)$*, *$q^{\prime }=q_{s}$*, *$\in^{\prime }=\in$.

**Proof**. Suppose that there exists an adversary ***A*** which succeeds with advantage ∈. We built an algorithm ***B*** to play the forgeability game against the DVSSAgg signature scheme. Given the challenge public key $\mathrm{Pk}=(\;P,G,G_{r},e,X,Y$), the interaction between Algorithm ***B*** and adversary ***A*** is as follows:

**Setup. *B*** First, get Pk = ($\;P,G,G_{r},e,X,Y$) of the challenger, then set the public parameter $Pa=\left(P,G,G_{r},e,g,Y\right)$, public key $PK^{*}=X$*,* initializes the list of keys and sets it to an empty set.
**Certification Queries.** The adversary ***A*** provides a key pair $\left(PK_{i},SK_{i}\right)$ and adaptability requires public key authentication. ***B*** checks the validity of the key pair and adds it to the key list.
**Signature Queries.** The adversary ***A*** provides the message chain $\bar{M^{\prime }}=\left(M_{1},M_{2},\ldots \ldots M_{k}\right)$, the sequential aggregate signature $\sigma^{\prime }$ under the public key $PK^{’}=\left(X_{1},X_{2},\ldots \ldots ,X_{k}\right)$ and a new message M and public key $PK^{*}$, then ***B*** execute the signature query as follows:
  (1) Check the validity of the signature $\sigma^{\prime }$, and check that each component of $PK^{’}=\left(X_{1},X_{2},\ldots \ldots ,X_{k}\right)$ exists in the key list.
  (2) Ask the signature oracle to get the σ of M, and the signed public keys is $PK^{*}$.
  (3) For each message $M_{i}$, run DVSSAggSign algorithm to get the sequential aggregate signature for the designated verifier σ about the message $\bar{M^{\prime }}||M$, and send it to ***A***.
**Output.** The adversary ***A*** outputs the forged sequential aggregate signature for the specific verifier $\sigma^{*}={\left(A^{*},B^{*},C^{*}\right)}^{D}$ about the message chain $\bar{M^{*}}=\left(M_{1},M_{2},\ldots \ldots M_{n}\right)$. The public key is $\bar{PK^{*}}=\left(PK_{1},PK_{2},\ldots \ldots ,PK_{n}\right)$. We assume that $PK_{1}=PK^{*}$, the forgery process of algorithm ***B*** is as follows:
  (1) ***B*** first runs the sequential aggregate validation algorithm for the designated verifier, then verifies the validity of the signature $\sigma^{*}$ and at the same time confirms that the challenge public key $PK^{*}$ must be in the $\bar{PK^{*}}$ and $M_{1}$ must not have been questioned by adversary ***A*** about the signature oracle.
  (2) In $\bar{PK^{*}}$, $PK_{i}=X_{i}$, retrieve the private key $SK_{i}=x_{i}$ from the key list $PK_{i}$, then compute:
    $$A=A^{*},\;B=B^{*},\;C=(C^{*}{\cdot ({\left(A^{*}\right)}^{\sum_{i=2}^{n}X_{i}}{\cdot (B^{*})}^{\sum_{i=2}^{n}X_{i}M_{i}})}^{-1}$$
  (3) Output the $\sigma^{*}={\left(A,B,C\right)}^{D}$ about $M^{*}=M_{1}$.
    Furthermore, we can prove the correctness of $\sigma^{*}$ of $M_{1}$ which is forged by the algorithm ***B***:
    $$e\left(C,g\right)=e(C^{*}{\cdot ({\left(A^{*}\right)}^{\sum_{i=2}^{n}X_{i}}{\cdot (B^{*})}^{\sum_{i=2}^{n}X_{i}M_{i}})}^{-1},g)=\;e({\left(A^{*}\right)}^{\sum_{i=1}^{n}X_{i}}{\left(B^{*}\right)}^{\sum_{i=1}^{n}X_{i}M_{i}}{\cdot (A^{*})}^{-\sum_{i=2}^{n}X_{i}}{\cdot (B^{*})}^{-\sum_{i=2}^{n}X_{i}M_{i}},g)=\;e({\left(A^{*}\right)}^{X_{1}}{\cdot (B^{*})}^{X_{1}M_{1}},g)=\;e(A^{*},g^{X_{1}})\cdot \;e\left(B^{*},g^{x_{1}M_{1}}\right)=\;e\left(A,X\right)\cdot e\left(B,X^{M^{*}}\right)$$

 □

## 6. Experiment

**Figure 5:**
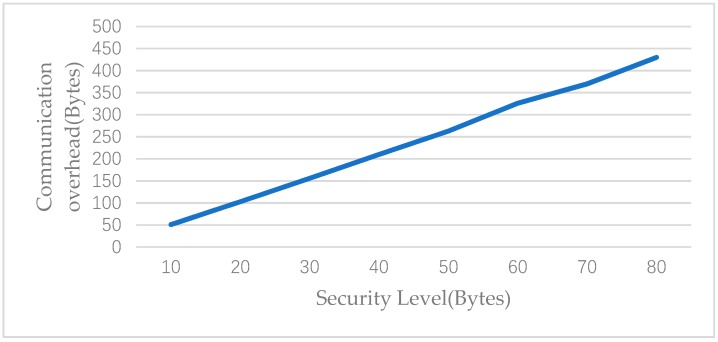
Communication overhead vs. security level.

Our main concern was the energy consumption for message computation and transmission. In terms of communication, signcryption is a major contributor to the communication overhead. That is to say, the communication overhead is mainly related to the size of the signed message. For a typical WBAN, it is sufficient to be 2 bytes for each user. In our evaluation, the bilinear e employs the Tate pairing. The elliptic curve is defined over $F_{p}$. The order q of $G_{1}$ and $G_{2}$ is a 20-byte prime. In order to deliver a level of security equivalent to that of 1024-bit RSA algorithm, p should be a 64-byte prime if $G_{2}$ is a q-order subgroup of the multiplicative group of the finite field $F_{P^{2}}$. In the following analysis, we set p to be 30 bytes in length for the finite field $F_{P^{3}}$. The overhead in terms of p is 5|p| + 4 for signcryption and 1 for decryption. Figure 5 illustrates the relationship between communication overhead and security levels. We note that the communication overhead increases as the security level increases. 

Furthermore, we used the method proposed in [42] to evaluate energy consumption in DVSSA. As shown in [43], a Chipcon CC1000 radio used in Crossbow MICA2DOT motes consumes 28.6 μJ and 59.2 μJ to respectively receive and transmit one byte. For our DVSSA scheme, the total message size is 30 bytes, leading to a total energy consumption (on both transmitting and receiving messages) of (5|p| + 4) ∗ (28.6 + 59.2) μJ = (0.439|p| + 0.3512) mJ for one user. When there are W users, the total energy consumption on communications is (W ∗ (0.439|p| + 0.3512)) mJ. We report the comparative results between DVSSA and the baseline approaches proposed in [42] on energy consumption in Table 2. Note that to evaluate the energy consumptions of the baseline approaches that make use of broadcasting, we adopted the model in [42].

**Figure 6:**
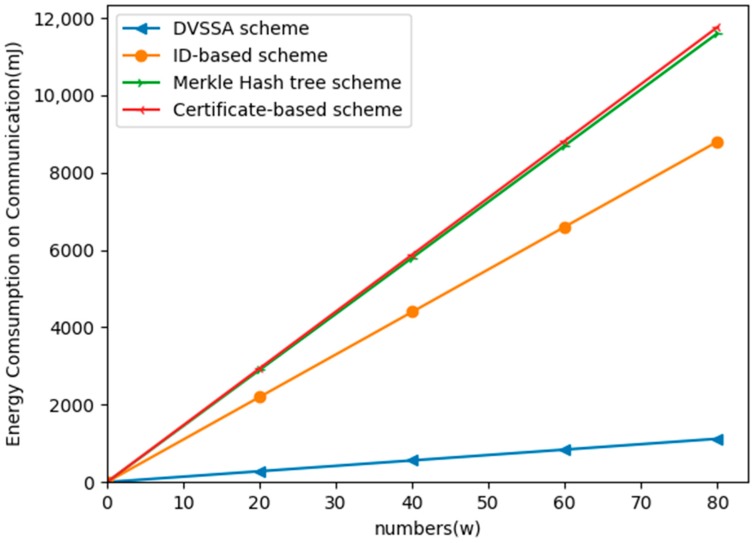
Energy consumption on communications with regard to the number of users.

Figure 6 shows the energy consumption on the communication as a function of the number of users. As can be seen from the figure, DVSSA consumes much less energy than the Merkle hash tree-based scheme, certificate-based scheme, and ID-based scheme [42].

**Figure 7:**
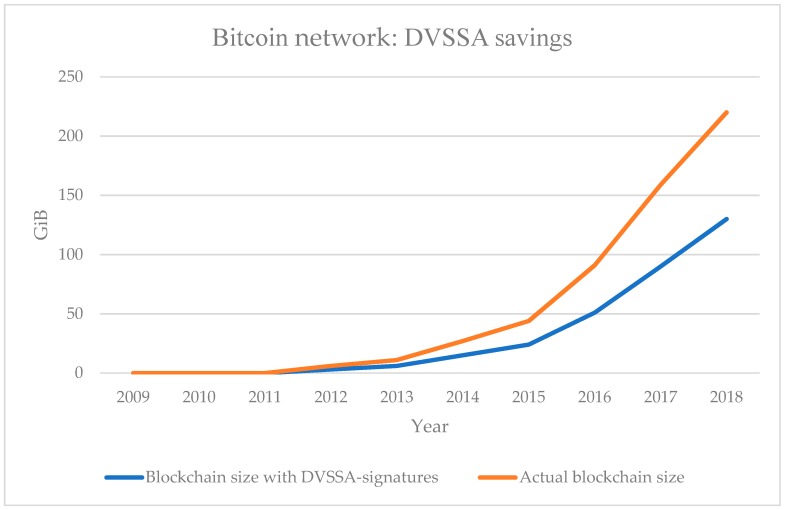
Size of the Bitcoin blockchain with and without DVSSA signatures.

Lastly, to analyze the impact of the sequential aggregate signature scheme with a designated verifier, we simulated the historical blockchain of bitcoin to determine whether our scheme has real potential to save space. Figure 7 shows the cumulative blockchain size to date, and what the blockchain size would be if all transaction signatures were replaced with an ordered aggregation signature for each transaction with only one specified verifier. Note that this includes only the overhead saved by using the ordered aggregation signature of the specified verifier, not the overhead saved by public key aggregation.

## 7. Conclusions

In this paper, we propose a data storage mechanism based on blockchain with privacy protection in a wireless body area network. In one hand, we designed a sequential aggregate signature scheme with a designated verifier which ensures the user’s data can only be viewed by the administrator and compresses the size of the blockchain storage space. When in other hands, we store the data in the blockchain through the blockchain technology. Through the tamper resistance characteristic of the blockchain, the integrity of the user data is guaranteed. Through experiments, we found that using our signature can compress the storage space of the block chain and achieves the purpose of saving resources. The novelty of our proposed method is mainly reflected in the use of blockchain as the storage space. In addition, we use digital signatures to ensure the security of the data collected in the WBAN. Finally, we also use Cloud technology for the intermediate transition.

## Figures and Tables

**Table 1:** Security threats and solutions in the WBAN.

Security Threats:	Security Requirements	Possible Solutions
Unverified or unauthorized access	Verified or authorized access	Random key distribution Public key encryption
Information leakage	Confidentiality	Link layer or network layer encryption Access control
Tampering with message	Integrity	Type a secure hash function A digital signature
Denial-of-service attack (DoS)	Usability	Intrusion detection Redundant routing
Node capture, damaged nodes	The resilience of the damaged node	Consistency checking and node undo tamper-proof
Routing attacks	Secure Routing	Security routing protocol
Intrusions and advanced security attacks	Security group management, intrusion detection	Secure group communication, intrusion detection

**Table 2:** Energy consumption on communications.

The Schemes	Total Size	Energy Consumption (mJ)
DVSSA scheme	P = 30 bytes	13.52 W
Certificate-based scheme	N = 512	146.99 W
Merkle hash tree scheme	N = 512	144.56 W
ID-based scheme	N = 512	111.02 W
